# Supplementary material for: FERN – a Java framework for stochastic simulation and evaluation of reaction networks
Source: BMC Bioinformatics. 2008 Aug 29;9:356. doi: 10.1186/1471-2105-9-356 (PMC2553347; doi:10.1186/1471-2105-9-356)
Supplement: Additional file 1 — FERN distribution, Version 1.3. This archive contains the FERN source code and binaries as well as documentation and example models in FernML and SBML. [file 1471-2105-9-356-S1.zip › fern/doc/javadoc/index-files/index-16.html]

R-Index


---


|  |  |  |
| --- | --- | --- |
| **Overview** \| Package \| Class \| Use \| **Tree** \| **Deprecated** \| **Index** \| **Help** | |  |
| **PREV LETTER**   **NEXT LETTER** | **FRAMES**    **NO FRAMES**     **All Classes** |  |


A B C D E F G H I J L M N O P R S T U V 

---


## **R**

**RandomNumber** - Class in fern.benchmark: Benchmarking of the time needed for different random number generations. **RandomNumber()** - Constructor for class fern.benchmark.RandomNumber: **RandomNumberGeneratorCallObserver** - Class in fern.simulation.observer: Observes the calls to the random number generator and count the number of drawn random number of different distributions. **RandomNumberGeneratorCallObserver(Simulator)** - Constructor for class fern.simulation.observer.RandomNumberGeneratorCallObserver: Creates the observer for the given simulator. **reactantHistos** - Variable in class fern.simulation.algorithm.AbstractBaseTauLeaping: **reactionDiscovered(int)** - Method in interface fern.analysis.NetworkSearchAction: Gets called when a reaction is inserted into the search structure. **reactionFinished(int)** - Method in interface fern.analysis.NetworkSearchAction: Gets called when a reaction gets out of the search structure. **ReactionIntervalObserver** - Class in fern.simulation.observer: Observes number of firings of reactions repeatedly after certain intervals. **ReactionIntervalObserver(Simulator, double, String...)** - Constructor for class fern.simulation.observer.ReactionIntervalObserver: Creates the observer for a given simulator, a given interval and given neighbors. **ReactionIntervalObserver(Simulator, double, int...)** - Constructor for class fern.simulation.observer.ReactionIntervalObserver: Creates the observer for a given simulator, a given interval and given reaction indices. **readNetwork(File)** - Static method in class fern.network.NetworkLoader: Tries to read the given file and returns the network in it (if there is one) **registerEvents(Simulator)** - Method in class fern.network.sbml.SBMLNetwork: Registers the `SBMLEventHandlerObserver` for each event in the sbml file to the Simulator. **registerNewTheta(Observer, double)** - Method in class fern.simulation.Simulator: Is called by `Observer.setTheta(double)`. **reinitialize()** - Method in class fern.simulation.algorithm.GibsonBruckSimulator: **reinitialize()** - Method in class fern.simulation.algorithm.GillespieEnhanced: **reinitialize()** - Method in class fern.simulation.algorithm.GillespieSimple: **reinitialize()** - Method in class fern.simulation.algorithm.HybridMaximalTimeStep: **reinitialize()** - Method in class fern.simulation.Simulator: Reset propensities when a event has been executed. **reloadNetwork()** - Method in class fern.cellDesigner.ui.MainFrame: **reloadNetwork(NetworkChecker)** - Method in class fern.cytoscape.ui.MainFrame: **reloadNetworkByButton()** - Method in class fern.cytoscape.ui.MainFrame: **resetAmount()** - Method in class fern.network.AmountManager: Resets the amount of each species to the initial amount retrieved by the networks `AnnotationManager`. **resetColors()** - Method in class fern.cytoscape.FernVisualStyle: **resetCounts()** - Method in class fern.tools.Stochastics: Resets the counts for each distribution to zero. **resetSeed()** - Method in class fern.tools.Stochastics: Sets the seed to the current date **REVERSIBLE\_SUFFIX** - Static variable in class fern.network.modification.ReversibleNetwork: Suffix which marks annotations for virtually created reverse reactions. **ReversibleNetwork** - Class in fern.network.modification: Doubles each reaction in a way that each original unidirectional reaction becomes reversible.As proposed by `ModifierNetwork`, the reactions are not copied but the indices are redirected. **ReversibleNetwork(Network, PropensityCalculator)** - Constructor for class fern.network.modification.ReversibleNetwork: Creates a new network from an original network and virtually creates for each reaction a new inverse reaction. **RND** - Static variable in class fern.simulation.algorithm.IndexedPriorityQueue: **rollback()** - Method in class fern.network.AmountManager: Restore the amount array from the recently saved one.

---


|  |  |  |
| --- | --- | --- |
| **Overview** \| Package \| Class \| Use \| **Tree** \| **Deprecated** \| **Index** \| **Help** | |  |
| **PREV LETTER**   **NEXT LETTER** | **FRAMES**    **NO FRAMES**     **All Classes** |  |


A B C D E F G H I J L M N O P R S T U V 

---
